# *Pseudomonas aeruginosa* Induced Airway Epithelial Injury Drives Fibroblast Activation: A Mechanism in Chronic Lung Allograft Dysfunction

**DOI:** 10.1111/ajt.13690

**Published:** 2016-02-26

**Authors:** L. A. Borthwick, M. I. Suwara, S. C. Carnell, N. J. Green, R. Mahida, D. Dixon, C. S. Gillespie, T. N. Cartwright, J. Horabin, A. Walker, E. Olin, M. Rangar, A. Gardner, J. Mann, P. A. Corris, D. A. Mann, A. J. Fisher

**Affiliations:** ^1^ Tissue Fibrosis and Repair GroupInstitute of Cellular MedicineNewcastle UniversityNewcastle upon TyneUK; ^2^ School of Mathematics and StatisticsNewcastle UniversityNewcastle upon TyneUK; ^3^ Institute of TransplantationNewcastle Upon Tyne Hospitals NHS Foundation TrustFreeman HospitalNewcastle upon TyneUK

## Abstract

Bacterial infections after lung transplantation cause airway epithelial injury and are associated with an increased risk of developing bronchiolitis obliterans syndrome. The damaged epithelium is a source of alarmins that activate the innate immune system, yet their ability to activate fibroblasts in the development of bronchiolitis obliterans syndrome has not been evaluated. Two epithelial alarmins were measured longitudinally in bronchoalveolar lavages from lung transplant recipients who developed bronchiolitis obliterans syndrome and were compared to stable controls. In addition, conditioned media from human airway epithelial cells infected with *Pseudomonas aeruginosa* was applied to lung fibroblasts and inflammatory responses were determined. Interleukin‐1 alpha (IL‐1α) was increased in bronchoalveolar lavage of lung transplant recipients growing *P. aeruginosa* (11.5 [5.4–21.8] vs. 2.8 [0.9–9.4] pg/mL, p < 0.01) and was significantly elevated within 3 months of developing bronchiolitis obliterans syndrome (8.3 [1.4–25.1] vs. 3.6 [0.6–17.1] pg/mL, p < 0.01), whereas high mobility group protein B1 remained unchanged. IL‐1α positively correlated with elevated bronchoalveolar lavage IL‐8 levels (r^2^ = 0.6095, p < 0.0001) and neutrophil percentage (r^2^ = 0.25, p = 0.01). Conditioned media from *P. aeruginosa* infected epithelial cells induced a potent pro‐inflammatory phenotype in fibroblasts via an IL‐1α/IL‐1R‐dependent signaling pathway. In conclusion, we propose that IL‐1α may be a novel therapeutic target to limit *Pseudomonas* associated allograft injury after lung transplantation.

## Abstract 2

This study demonstrates that a *Pseudomonas aeruginosa* challenge of lung epithelial cells causes the release of IL‐1α and the induction of an inflammatory phenotype in lung fibroblasts, and provides evidence of an association between infection with *P. aeruginosa,* IL‐1α levels in bronchoalveolar lavage fluid, and the development of bronchiolitis obliterans syndrome following lung transplantation.

**Abbreviations**
BAL: bronchoalveolar lavage
BOS: bronchiolitis obliterans syndrome
CLAD: chronic lung allograft dysfunction
DAMPs: damage‐associated molecular patterns
HMGB1: high mobility group protein B1
IL‐1α: interleukin‐1 alpha
LDH: lactate dehydrogenase
PLEC: primary human lung epithelial cells
PLF: primary human lung fibroblasts
RAS: restrictive allograft syndrome

## Introduction

Chronic lung allograft dysfunction (CLAD) causes significant loss of function to the transplanted lung and is the major factor limiting long‐term survival after lung transplant 1. When this process affects the small‐ and medium‐sized airways resulting in airflow limitation, it is referred to as bronchiolitis obliterans syndrome (BOS). Histologically, BOS is characterized by progressive loss of bronchial epithelium, neutrophil influx, chronic inflammation, and fibroproliferation causing small airway obliteration 1, 2, 3, 4. BOS affects approximately 50% of patients within 5 years of transplantation and accounts for 30% of deaths occurring within 3 years 5.

BOS is a progressive condition that is often accompanied by repeated episodic or chronic lower respiratory tract infection 6, 7, 8 and numerous studies have shown that bacterial infection, specifically the gram‐negative organism *Pseudomonas aeruginosa*, significantly increase the risk of developing BOS in posttransplant and nontransplant patients 6, 9, 10, 11, 12, 13, 14. Chronic *P. aeruginosa* infection can lead to persistent inflammation and damage of the respiratory epithelium 15, 16, and damaged epithelial cells may be a source of danger signals referred to as alarmins or damage‐associated molecular patterns (DAMPs) 17, 18, 19. Alarmins are a diverse class of molecules serving many important intracellular functions including maintaining chromatin structure (high mobility group protein B1 [HMGB1]), regulating protein folding (heat shock proteins), or modulating gene expression (interleukin‐1 alpha [IL‐1α]) 20, 21.

Chronic inflammation and progressive fibroproliferation are two characteristic features of BOS 22. Until quite recently, fibroblasts have been regarded as cells whose role was limited to the restoration of tissue architecture during physiological wound repair or deposition of various components of extracellular matrix in pathological repair. However, emerging evidence suggests that fibroblasts may also function as important mediators of innate immune responses and may act to modulate the switch from acute to chronic inflammation, processes that coexist in fibrosis 23.

IL‐1α is a member of the interleukin‐1 superfamily, which consists of 11 cytokines including IL‐1β, IL‐18, and IL‐33. IL‐1α has been shown to be elevated in the lungs of chronic obstructive pulmonary disease patients 24, and recent work has identified anti‐IL‐1α autoantibodies in the blood of idiopathic pulmonary fibrosis patients 25 and increased levels of IL‐1Ra in lung allograft recipients who developed BOS 26, suggesting that extracellular IL‐1α may be an undesirable and potentially harmful factor in fibrotic lung diseases. Immune cells such as monocytes and macrophages actively transcribe and release IL‐1α in response to a range of stimuli including metal particles 27 and bacterial products such as lipopolysaccharide 28. In contrast, IL‐1α is synthesized and constitutively stored in the cytoplasm of epithelial cells. Recent studies have revealed that damaged epithelial cells can trigger a proinflammatory phenotype in lung fibroblasts through the release of DAMPs, specifically IL‐1α 19. Epithelial damage may be a result of infection with bacteria 29 or virus 30 or may be caused by noninfective insults such as air pollution 31 **,** oxidative stress 32, aspiration injury 33, or cigarette smoke 24, 34. IL‐1β is also upregulated in chronic inflammatory lung diseases and may be released from activated macrophages 35.

Although IL‐1 family members are known to play a role in mediating the innate immune responses, the relationship between IL‐1α and other alarmins with *P. aeruginosa* infection in the development of BOS after lung transplantation has not been investigated. Therefore, in this study we investigate the relevance of selected alarmins in BOS and investigate whether *P. aeruginosa* induced epithelial cell damage can drive fibroblast activation and in particular an inflammatory phenotype in lung fibroblasts.

## Materials and Methods

This study was approved by the Newcastle and North Tyneside Local Regional Ethics Committee (2001/179), and informed written consent was obtained from all study patients.

### Bronchoalveolar lavage

All patients had undergone surveillance bronchoscopic evaluation at approximately 1, 3, 6, and 12 months posttransplant and at the time BOS was suspected or diagnosed. A standardized 180 mL bronchoalveolar lavage (BAL) comprising three sequentially administered and suctioned 60 mL washes of saline was performed from a subsegment of the right middle or lower lobe or left lower lobe (n = 117 from patients who developed BOS, n = 91 from patients who remained stable). An aliquot was sent for microbiological culture including extended testing for bacterial and fungal pathogens. The remaining sample was filtered and the cellular fraction was separated by centrifugation. Total and differential cell counts were performed and the supernatant was stored for analysis.

### MSD electrochemiluminescence assay

IL‐1α and IL‐8 concentrations in BAL were measured using MSD electrochemiluminescence detection kits (Meso Scale Discovery, Gaithersburg, MD).

### Cell and bacterial culture

See Data S1 Supplementary Materials and Methods in the Supporting Information.

### Epithelial cell treatments

Live *P. aeruginosa* or heat‐killed *P. aeruginosa* were added to primary lung epithelial cells (PLEC) (see Data S1 Supplementary Materials and Methods for details of isolation and culture conditions) at 10^4^ to 10^8^ cfu/mL. After 12 h the media (hereafter referred to as conditioned media) was harvested and filtered (0.2 μm) to remove all bacteria.

### Fibroblast treatments

Primary lung fibroblasts (PLF) (see Data S1 Supplementary Materials and Methods for details of isolation and culture conditions) were treated with conditioned media from *P. aeruginosa* challenged PLEC that had been pre‐incubated for 1 h with IL‐1α or IL‐1β neutralizing antibody (both 4 μg/mL) (R&D Systems, Minneapolis, MN). To inhibit IL‐1R signaling, PLF were pretreated for 1 h with IL‐1Ra (500 ng/mL) (R&D Systems) after which the culture media was replaced with conditioned media containing the same dose of IL‐1Ra. After 24 h, media was collected for enzyme‐linked immunosorbent assay (ELISA) analysis. Cells were harvested into NucleoSpin^®^ RNA lysis buffer (Macherey‐Nagel, Duren, Germany) for RNA extraction.

PLF were pretreated with dexamethasone or azithromycin (0.31–5 μM) for 1 or 24 h, respectively, before addition of recombinant human IL‐1α (100 pg/mL) or conditioned media from PLEC challenged with *P. aeruginosa*. IL‐1α was blocked by pre‐incubating the conditioned media for 1 h with IL‐1α (4 μg/mL) neutralizing antibody. After 24 h, media was collected for ELISA analysis.

### LDH assay

Lactate dehydrogenase (LDH) release was assessed as a measure of cellular cytotoxicity using the Pierce LDH Cytotoxicity Assay Kit (Thermo Scientific, Waltham, MA).

### ELISA

IL‐1α, IL‐1β, IL‐6, and IL‐8 concentrations in cell culture supernatants were measured using DuoSet^®^ kits (R&D Systems). HMGB1 was measured with a direct ELISA (see Data S1 Supplementary Materials and Methods).

### PCR

RNA extraction was performed using Nucleospin RNA II kit (Macherey‐Nagel). Quantitative reverse transcription polymerase chain reaction (qPCR) was performed using AffinityScript^™^ Multiple Temperature RT kit (Agilent Technologies, Santa Clara, CA). Relative gene expression was measured using SYBR^®^ Green JumpStart^™^ Taq ReadyMix^™^ (Sigma, St Louis, MO). Primers for qPCR are listed in Supplementary Table S1.

### Immunohistofluorescence

Lung tissue sections (5 μm) were deparaffinized in xylene, rehydrated in graded alcohol, and antigen retrieval was performed in EDTA (1 mM, pH 8.0) for 10 min in a microwave. Samples were blocked in 1% bovine serum albumin (BSA) and incubated overnight at 4°C with anti‐IL‐1α (AF‐200‐NA, R&D) or an IgG1 control antibody (both 10 μg/mL, 1% BSA). Slides were washed and incubated with a fluorescein isothiocyanate–conjugated anti‐goat secondary antibody (F7367, Sigma) for 2 h at room temperature. Slides were washed and mounted in VectorShield mounting media containing DAPI (Vector Laboratories, Burlingame, CA). Images were acquired using a Leica TCS SP2 UV confocal microscope.

### Statistical analysis

Mean log_10_ of IL‐1α, IL‐8, and HMGB1 concentration and neutrophil number and percentage in BAL samples per patient were used for analysis. Additional stratification of patient BAL samples were performed to compare differences in BAL samples collected <3 months before or after BOS diagnosis versus BAL samples collected >3 months before or after BOS diagnosis (Figures 2 and S1) and in patients with culture positive (any organism or *P. aeruginosa*) compared to culture negative BAL samples (Figures 3, S2, and S5).

Changes in log_10_ of IL‐1α and HMGB1 concentration and neutrophil number and percentage in BAL samples collected longitudinally were assessed using a random‐effects model, with patients as the random component (Figures 2, 3, S1, S3, and S4). All p‐values relate to the gradient of the fitted line. To plot an average line, we took the mean value at T0 (BOS only–time of BOS diagnosis taken as T0) or T6 (stable only–time of transplant was taken as T0) as the y‐intercept.

Data were analyzed using chi‐square test for trend test, paired Student's t‐test for normally distributed data, or Mann–Whitney U test for non‐normally distributed data as appropriate. Significance was defined by p < 0.05. Results are presented as median (Figures 2, 3, S1, S2, and S5) or mean (standard error of the mean) (Figures 4, 5, and 6).

## Results

### Patient characteristics

**Figure 1:**
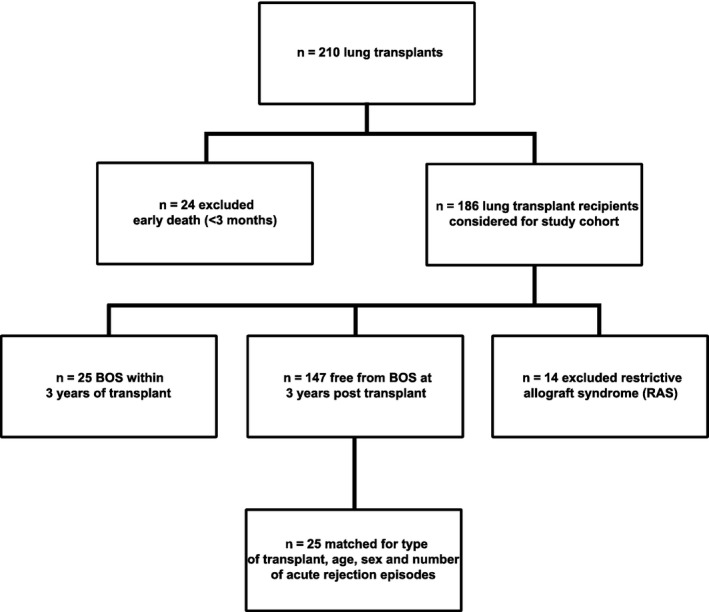
**Patient selection.** All lung transplant patients (n = 210) from March 2005 to February 2010 were considered for inclusion in the retrospective study cohort. n = 24 were excluded due to early death (within 3 months of transplantation) and n = 14 were excluded for development of restrictive allograft syndrome (RAS) within 3 years of transplantation. Of the remaining patients, n = 25 (13.4%) developed BOS within 3 years of transplantation and were selected as our study population. n = 147 remained free from BOS at 3 years, and n = 25 were selected as a case‐controlled group. The nested case–control group was matched for type of transplant, age, sex, and number of acute rejection episodes. BOS, bronchiolitis obliterans syndrome.

Our transplant program performed 210 lung transplants from 2005 to 2010. Patients were excluded from our retrospective study due to early death (within 3 months of transplantation) (n = 24) and for development of restrictive allograft syndrome (RAS) within 3 years of transplantation (n = 14). Of the remaining patients, n = 25 (13.4%) developed BOS within 3 years of transplantation. BOS was diagnosed in accordance with International Society for Heart and Lung Transplantation guidelines 36. All patients demonstrated persistent obstructive spirometry with an FEV_1_ of <80% of their posttransplant baseline. All had acute rejection or active infection excluded at bronchoscopy as the explanation for their graft dysfunction and had high‐resolution computed tomography lung imaging consistent with BOS and with no evidence of parenchymal changes of potential RAS. A nested case‐controlled group of n = 25 patients was identified from the n = 147 lung transplant recipients who remained stable at 3 years posttransplant. The limited size of the whole control cohort meant it was not possible to individually pair each case with a control adequately for type of transplant, age, sex, and number of acute rejection episodes and therefore the approach adopted was for the control group as a whole to have an age, sex, and acute rejection history profile as close as possible to the cases. The controls were initially identified by closest age to the cases and then gender and finally acute rejection episodes. Controls were then removed to improve the matching of the overall control group to the cases until a final n = 25 were identified (Figure 1 and Table 1).

**Table 1:** Patient demographics

	BOS	Non‐BOS	p‐value
Number of patients	25	25	
Median age (years)	46 (23–63)	45 (19–64)	p = 0.47
Sex distribution			
Male	15	20	p = 0.12
Female	10	5	
Number of acute rejection episodes	22	21	p = 0.35
Median number of BAL samples per patient	5 (2–8)	3 (2–4)	p < 0.001

Underlying condition	BOS		Non‐BOS		p‐value
	Number	%	Number	%	
COPD	9	36%	2	8%	p = 0.37
Cystic fibrosis	6	24%	11	44%	
Fibrotic lung disease	5	20%	8	32%	
α1‐Anti‐trypsin deficiency	2	8%	2	8%	
Asthma	1	4%	0	0%	
Histiocytosis	1	4%	0	0%	
Lymphangioleiomyomatosis	1	4%	0	0%	
Primary pulmonary hypertension	0	0%	1	4%	
Bronchiectasis	0	0%	1	4%	

Type of transplant	BOS		Non‐BOS		p‐value
	Number	%	Number	%	
Single lung	9	36%	7	28%	p = 0.40
Bilateral lung	16	64%	17	68%	
Heart lung	0	0%	1	4%	

Organisms cultured from BAL	BOS		Non‐BOS		p‐value
	Number	%	Number	%	
*Pseudomonas aeruginosa*	5	20%	7	28%	p = 0.25
*Candida albicans*	9	36%	11	44%	
*Aspergillus fumigatus*	5	20%	0	0%	
*Proteus mirabilis*	1	4%	0	0%	
*Stenotrophomonas maltophilia*	3	12%	0	0%	
*Staphylococcus aureus*	2	8%	3	12%	
*Acinetobacter baumannii*	1	4%	0	0%	
*Aspergillus nidulans*	1	4%	0	0%	
*Enterobacter cloacae*	1	4%	0	0%	
*Haemophilus influenzae*	1	4%	0	0%	
*Klebsiella pneumonia*	1	4%	0	0%	
*Serratia* spp	1	4%	1	4%	
*MRSA*	0	0%	3	12%	
*Escherichia coli*	0	0%	2	8%	
*Burkholderia cepacia* complex	0	0%	1	4%	
*Exophiala* spp	0	0%	1	4%	
No organisms cultured	11	44%	5	20%	

BAL, bronchoalveolar lavage; BOS, bronchiolitis obliterans syndrome; COPD, chronic obstructive pulmonary disease; MRSA, methicillin‐resistant *Staphylococcus aureus*.

There was no significant difference in the type or number of organisms cultured from BAL (Tables 1, S2, S3, and S4); however, there was a significant difference in the number of BAL samples per patient, with more samples collected from the BOS group compared to the stable group (p < 0.001) (Table 1). This was primarily due to more symptom‐driven bronchoscopies, in addition to the surveillance bronchoscopies, in the BOS group.

### IL‐1α is elevated in BAL of patients with posttransplant BOS

**Figure 2:**
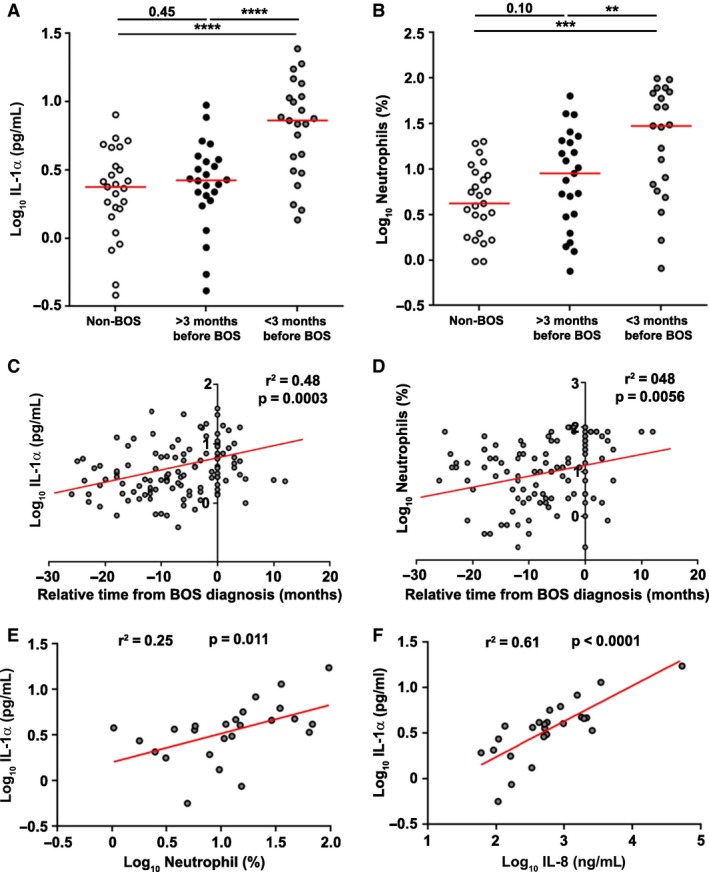
**Increased neutrophilia and elevated levels of IL‐1α in BAL of posttransplant patients at the time of BOS diagnosis.** Mean IL‐1α (A) concentrations and neutrophil percentage (B) in BAL of lung transplant recipients who remained stable at 3 years (n = 25) or develop BOS within 3 years of transplant (n = 25). BALs from patients who developed BOS were grouped into BAL samples taken more than 3 months before or after BOS diagnosis (>3 months before BOS) and BAL samples taken <3 months before or after BOS diagnosis (<3 months before BOS). Data were analyzed using Mann–Whitney U test or paired t‐tests as appropriate and are presented as median. Correlation between the relative time from BOS diagnosis and IL‐1α concentration (C) and neutrophil percentage (D) in BAL samples from patients who develop BOS. Data were analyzed using a multiple linear regression model with varying intercept. All p‐values relate to the gradient of the fitted line. To plot an average line, we took the mean value at T0 (time of BOS diagnosis) as the y‐intercept. Correlation is shown between IL‐1α concentration and neutrophil percentage (E) and between IL‐1α and IL‐8 concentrations (F) in BAL samples from patients who develop BOS. Data were analyzed using a linear regression model. **p < 0.01, ***p < 0.001, ****p < 0.0001. BAL, bronchoalveolar lavage; BOS, bronchiolitis obliterans syndrome.

To evaluate the clinical relevance of alarmins in the transplanted lung, IL‐1α and HMGB1 concentration and the percentage and number of neutrophils were measured in BAL fluid collected longitudinally from n = 25 lung transplant recipients who developed BOS within 3 years of transplant and a nested case‐controlled group of n = 25 patients who remained stable. BAL samples collected more than 3 months before or after BOS diagnosis (>3 months before BOS) and BAL samples collected <3 months before or after BOS diagnosis (<3 months before BOS) were grouped. HMGB1 was not significantly different between BAL samples collected more than 3 months before BOS diagnosis and BAL samples collected within 3 months of BOS diagnosis (p = 0.60) (Figures S1A and B). However, IL‐1α was significantly higher in BAL collected within 3 months of BOS diagnosis compared to BAL collected more than 3 months before BOS diagnosis (p < 0.0001) (Figures 2A and S1A). Additionally, there was an increase in the percentage and number (both p < 0.01) of neutrophils in BAL collected within 3 months of BOS diagnosis compared to BAL collected more than 3 months before BOS diagnosis (Figures 2B and S1A and C). Moreover, IL‐1α concentration (r^2^ = 0.48, p = 0.0003) and neutrophil percentage (r^2^ = 0.48, p = 0.0056) (Figures 2C and D), but not HMGB1 concentration (r^2^ = 0.34, p > 0.99) (Figure S1D), showed an increase towards the time of BOS diagnosis. Finally, there was a positive correlation between the percentage of neutrophils (r^2^ = 0.25, p = 0.011) and IL‐8 levels (r^2^ = 0.61, p < 0.0001) (Figures 2E and F) with IL‐1α concentration in BAL of patients who develop BOS. These observations are in agreement with previous reports showing the association between neutrophilia and BOS development 37, 38.

### IL‐1α levels are increased in BAL from patients with positive organism cultures

**Figure 3:**
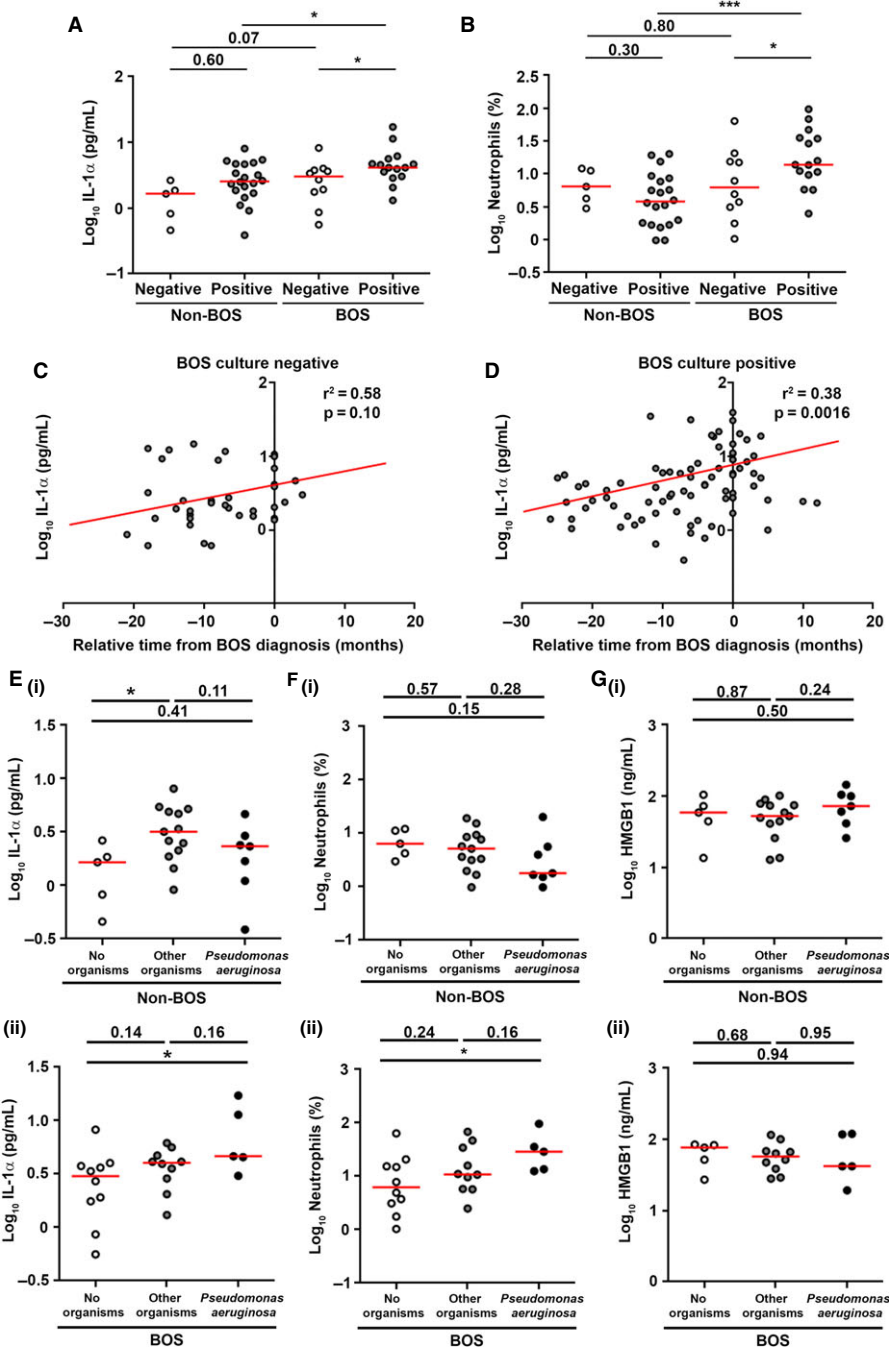
**IL‐1α and neutrophilia are elevated in** ***Pseudomonas aeruginosa*** **positive BAL in patients with BOS.** Mean IL‐1α (A) concentration and neutrophil percentage (B) in culture positive (any organism) and culture negative (no organisms) BAL samples from patients (n = 25) who developed BOS or remained stable (non‐BOS) (n = 25). Data were analyzed using Mann–Whitney U test and are presented as median. Correlation is shown between the relative time from BOS diagnosis and IL‐1α concentration in culture negative (n = 10) (C) and culture positive (n = 15) (D) BAL samples from patients who develop BOS. Data were analyzed using a multiple linear regression model with varying intercept. All p‐values relate to the gradient of the fitted line. To plot an average line, we took the mean value at T0 (time of BOS diagnosis) as the y‐intercept. Mean IL‐1α concentration (E), neutrophil percentage (F), and HMGB1 concentration (G) in culture negative (no organisms), culture positive for any organism other than *P. aeruginosa* (other organisms), and culture positive for *P. aeruginosa* (*P. aeruginosa*) BAL samples from patients who remained stable (non‐BOS) (i) and patients who developed BOS (ii). Data were analyzed using Mann–Whitney U test and are presented as median. *p < 0.05, ***p < 0.001. BAL, bronchoalveolar lavage; BOS, bronchiolitis obliterans syndrome; HMGB1, high mobility group protein B1.

We proceeded to investigate IL‐1α and HMGB1 levels and the number and percentage of neutrophils in BAL samples from BOS and non‐BOS patients without organisms cultured (culture negative) or with any positive organism culture (culture positive). IL‐1α was significantly increased in culture positive BAL compared to culture negative BAL (p < 0.05) in patients who develop BOS. In addition, IL‐1α was significantly increased in culture positive BAL from patients who develop BOS compared to culture positive BAL from non‐BOS patients (p < 0.05). Interestingly, a non–statistically significant trend towards an increase in IL‐1α concentration in BOS culture negative BAL compared to non‐BOS culture negative BAL (p = 0.07) was seen suggesting that, apart from bacterial or fungal infection, other factors driving IL‐1α release may be implicated in BOS (Figure 3A). BAL neutrophil percentage and number (both p < 0.05) were also increased in culture positive BAL compared to culture negative BAL in BOS patients. Similar to IL‐1α, BAL neutrophil percentage and numbers were significantly higher in BOS culture positive BAL compared to non‐BOS culture positive BAL (both p < 0.001) (Figures 3B and S2B). In contrast, no difference in HMGB1 levels was observed between groups (Figure S2A).

IL‐1α concentrations and neutrophilia in BAL presented as relative time from BOS diagnosis in culture positive and culture negative BAL samples demonstrated that there is a positive correlation between IL‐1α and time from BOS diagnosis in the BOS culture positive group (r^2^ = 0.38, p = 0.0016) and a non–statistically significant trend between IL‐1α and time from BOS diagnosis in the BOS culture negative group (r^2^ = 0.58, p = 0.10) (Figures 3C and D). In addition, there was a positive correlation between neutrophil percentage and time from BOS diagnosis in the BOS culture positive group (r^2^ = 0.41, p = 0.019) and a non–statistically significant trend between neutrophil percentage and time from BOS diagnosis in the BOS culture negative group (r^2^ = 0.50, p = 0.14) (Figure S3). Finally, no correlation was observed between IL‐1α or HMGB1 concentrations and neutrophil percentage or number relative to the number of months from transplantation in non‐BOS patients (Figure S4).

### IL‐1α levels are increased in *P. aeruginosa* positive BAL samples isolated from patients with posttransplant BOS

IL‐1α and neutrophil percentage were significantly increased in BAL positive for *P. aeruginosa* compared to culture negative BAL (both p < 0.05) in patients who developed BOS. No significant difference in IL‐1α or neutrophil percentage was seen between BAL positive for all other organisms and culture negative BAL in patients who developed BOS (p = 0.14 and p = 0.24, respectively). In contrast, no significant difference was seen in IL‐1α levels and neutrophil percentage in BAL positive for *P. aeruginosa* compared to culture negative BAL in non‐BOS patients (p = 0.41 and p = 0.15, respectively) (Figures 3E and F). Again, no difference in HMGB1 was observed (Figure 3G).

BOS and non‐BOS patients were divided into those with no positive BAL cultures, those with one positive BAL culture, and those with >1 positive BAL culture and IL‐1α and HMGB1 concentration and neutrophil percentage assessed. BOS patients with more than one positive culture BAL had significantly higher levels of IL‐1α and an increased percentage of neutrophils than BOS patients with no positive BAL cultures (both p < 0.05). In contrast, there was no significant difference in IL‐1α concentration or neutrophil percentage in non‐BOS patients (p = 0.053 and p = 0.33, respectively). No difference in HMGB1 was seen between groups in BOS and non‐BOS patients (Figure S5).

### *P. aeruginosa* infection of epithelial cells *in vitro* induced the release of epithelial alarmins including IL‐1α

**Figure 4:**
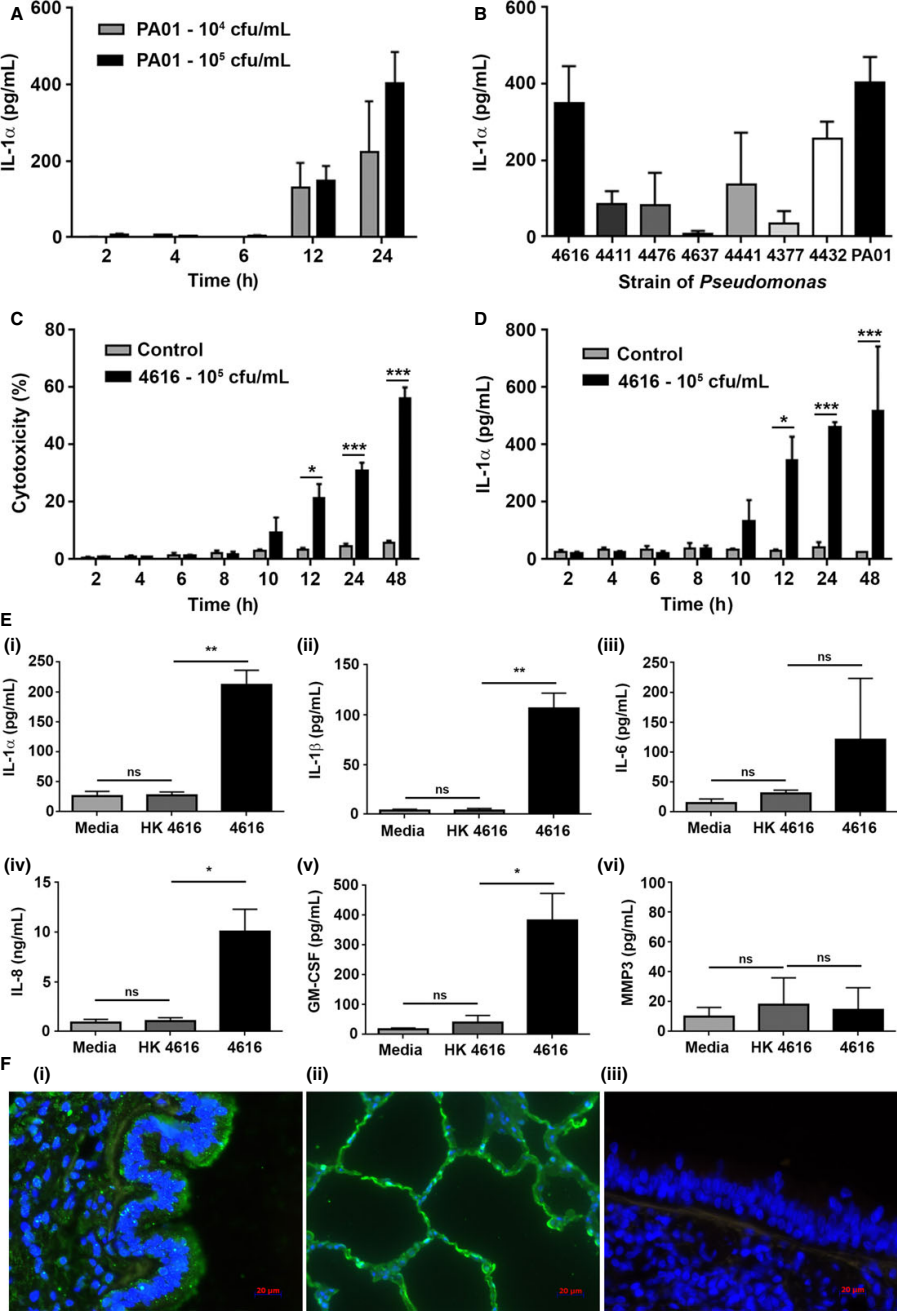
***Pseudomonas aeruginosa*** **promotes cell death and alarmin release in lung epithelial cells** ***in vitro.*** (A) Primary lung epithelial cells (PLEC) (n = 4) were challenged with *P. aeruginosa* (PA01) at 10^4^ or 10^5^ colony‐forming units (cfu)/mL for up to 24 h and IL‐1α release was measured. (B) PLEC (n = 4) were challenged with *P. aeruginosa* (PA01) and n = 7 strains of *P. aeruginosa* isolated from post–lung transplant patients at 10^5^ cfu/mL for 12 h and IL‐1α release was measured. PLEC (n = 4) were challenged with *P. aeruginosa* (4616) at 10^5^ cfu/mL for up to 48 h and cytotoxicity (release of lactate dehydrogenase) (C) and IL‐1α release (D) quantified. (E) PLEC (n = 4) were challenged with heat‐killed *P. aeruginosa* strain 4616 (HK4616), live *P. aeruginosa* strain 4616 at 10^5^ cfu/mL (4616) or media alone (media) for 12 h and the release/secretion of IL‐1α (i), IL‐1β (ii), IL‐6 (iii), IL‐8 (iv), GM‐CSF (v), and MMP3 (vi) quantified. Data were analyzed using paired t‐tests and presented as mean ± standard error of the mean. *p < 0.05, **p < 0.01, ***p < 0.001. (F) Lung tissue from patients with BOS was stained with an anti‐IL‐1α antibody to identify potential cellular sources of IL‐1α *in vivo*. IL‐1α protein is expressed in bronchial (i) and alveolar (ii) epithelium as well as other nonepithelial cell types. IgG1 control showed no staining in the epithelium (iii). DAPI was used as a nuclear counterstain. These data confirm that injured epithelium may be one of the potential sources of IL‐1α *in vivo*. BOS, bronchiolitis obliterans syndrome; DAPI, 4′,6‐diamidino‐2‐phenylindole; GM‐CSF, granulocyte‐macrophage colony‐stimulating factor; MMP3, matrix metalloproteinase 3.

To evaluate the association between bacterial infection, IL‐1α, neutrophilia, and BOS, we developed an *in vitro* model of *P. aeruginosa* infection of the airway epithelium and its subsequent effects on fibroblast activation. PLEC were initially infected with a laboratory reference strain of *P. aeruginosa* (PA01) at densities ranging from 10^4^ to 10^8^ cfu/mL for up to 24 h, and the release of IL‐1α was quantified. Infection of PLEC with PA01 at 10^4^ and 10^5^ cfu/mL for 12 and 24 h induced cell injury and some cell death (data not shown) and triggered the release of IL‐1α (Figure 4A). Consequently, a *P. aeruginosa* density of 10^5^ cfu/mL was used for all additional experiments. The effect of PA01 infection on cell viability (data not shown) and IL‐1α release was compared to clinical isolates of *P. aeruginosa* (n = 7) grown from the airway samples of posttransplant patients. The ability of the clinical strains to induce IL‐1α release was variable; however, *P. aeruginosa* strain 4616 (hereafter referred to as 4616) induced the strongest response and was used in subsequent experiments (Figure 4B). To confirm the suitability of 4616 for our experiments, we infected PLEC with 10^5^ cfu/mL for up to 48 h and measured cell injury and death as well as IL‐1α release (Figures 4C and D). Similar to the data generated with PA01, infection of PLEC with 4616 at 10^5^ cfu/mL induced cell injury, some cell death, and IL‐1α release at 12 h. Subsequent experiments were performed by infecting PLEC with strain 4616 at 10^5^ cfu/mL for 12 h.

PLEC isolated from n = 4 unused donor lungs were cultured for 12 h in media only (media), media containing heat‐killed 4616 (HK4616), or media containing live 4616 at 10^5^ cfu/mL (4616), and the release of epithelial alarmins (IL‐1α, HMGB1), inflammatory cytokines (IL‐1β, IL‐6, IL‐8), granulocyte‐macrophage colony‐stimulating factor (GM‐CSF), and matrix metalloproteinase 3 (MMP3) measured (Figure 4E). Challenge with live 4616 induced a significant increase in the release of IL‐1α (p < 0.01) (i), HMGB1 (data not shown), IL‐1β (p < 0.01) (ii), IL‐8 (p < 0.05) (iv), and GM‐CSF (p < 0.05) (v) but had no significant effect on IL‐6 (p = 0.44) (iii) and MMP3 (p = 0.91) (vi) compared to challenge with HK4616.

To answer the question of whether epithelial cells are a potential source of IL‐1α *in vivo*, we investigated the cellular localization of IL‐1α in lung tissue from patients with BOS. Our data show that airway epithelial cells (Figure 4Fi) and alveolar epithelial cells (Figure 4Fii) express IL‐1α and therefore are a potential source of IL‐1α *in vivo*. In addition, other nonepithelial cells are also positive for IL‐1α and therefore may contribute to the IL‐1α detected in BAL.

### IL‐1α released from *P. aeruginosa* infected epithelial cells induces fibroblast activation *in vitro*

**Figure 5:**
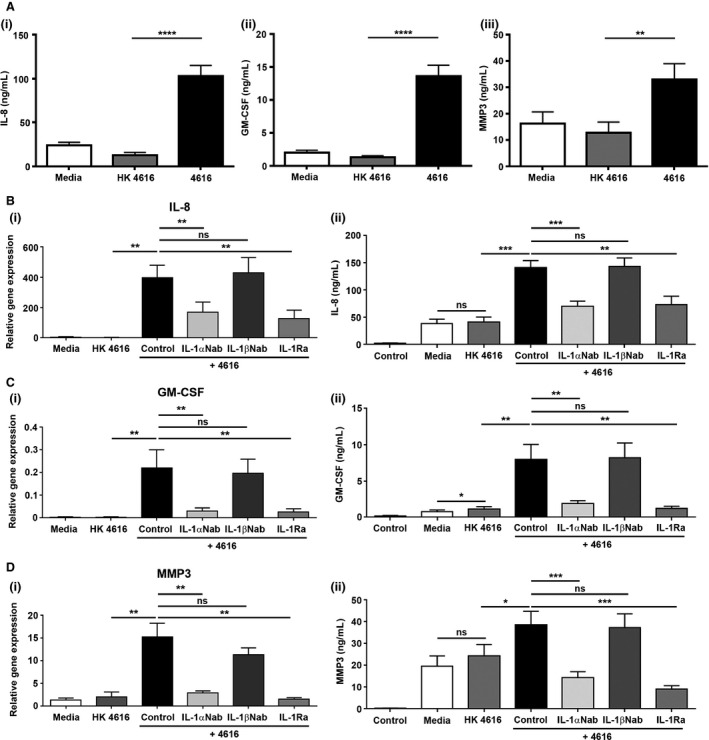
**IL‐1α is the primary epithelial alarmin driving fibroblast activation** ***in vitro.*** (A) Primary human lung fibroblasts (PLF, n = 4) were incubated with media from untreated primary human lung epithelial cells (PLEC, n = 4) (media), media from PLEC challenged with heat‐killed *Pseudomonas aeruginosa* strain 4616 (HK4616), and media from PLEC challenged with live *P. aeruginosa* strain 4616 at 10^5^ colony‐forming units/mL (4616). Protein secretion of IL‐8 (i), GM‐CSF (ii), and MMP3 (iii) was quantified. (B) To investigate the role of IL‐1α and IL‐1β, the media from PLEC challenged with live *P. aeruginosa* strain 4616 was preincubated for 1 h with IL‐1α (4 μg/mL) or IL‐1β (4 μg/mL)–neutralizing antibody. To investigate the role of the IL‐1R, PLF were pretreated for 1 h with IL‐1Ra (500 ng/mL) prior to the addition of conditioned media from PLEC. Relative gene expression (i) and protein secretion (ii) of IL‐8 (B), GM‐CSF (C), and MMP3 (D) were quantified. Data were analyzed using paired t‐tests and presented as mean ± standard error of the mean (A, n = 16; B–E, n = 8). *p < 0.05, **p < 0.01, ***p < 0.001, ****p < 0.0001. GM‐CSF, granulocyte‐macrophage colony‐stimulating factor; MMP3, matrix metalloproteinase 3.

Conditioned media from unchallenged PLEC (media), PLEC challenged with HK4616 for 12 h (HK4616), and PLEC challenged with live 4616 for 12 h (4616) (n = 4) was collected, filtered to remove all bacteria, and applied to PLF (n = 4). After 24 h, the media was harvested to investigate changes in protein secretion (Figure 5A). Conditioned media from PLEC challenged with HK4616 had little to no effect on IL‐8 (i), GM‐CSF (ii), and MMP3 (iii) secretion compared to conditioned media from unchallenged PLEC. In contrast, conditioned media from PLEC infected with live 4616 induced a significant increase in secretion of all inflammatory markers compared to conditioned media from PLEC challenged with HK4616 (all p < 0.01).

Previous work from our laboratory has demonstrated that PLF are induced to adopt a highly inflammatory phenotype in response to treatment with recombinant IL‐1α and IL‐1β but no effect is seen following treatment with recombinant HMGB1 17. Therefore, to investigate whether the IL‐1α or IL‐1β released from *P. aeruginosa* infected PLEC are involved in the activation of an inflammatory phenotype in PLF, we pre‐incubated the conditioned media from PLEC challenged with live 4616 with IL‐1α (IL‐1αNab) or IL‐1β (IL‐1βNab) neutralizing antibody for 1 h before adding to the PLF. In addition, PLF were pretreated with IL‐1Ra for 1 h before the conditioned media was added to investigate a role for the IL‐1 receptor in PLF activation. For all markers, neutralization of IL‐1α, but not IL‐1β, in the conditioned media or blocking the IL‐1R on PLF significantly reduced gene expression and protein secretion, suggesting that IL‐1α, signaling via IL‐1R, is the critical alarmin released from *P. aeruginosa* challenged epithelial cells involved in driving an inflammatory phenotype in fibroblasts (Figures 5B–D).

### Dexamethasone partially inhibits IL‐1α induced inflammatory responses in fibroblasts in vitro

**Figure 6:**
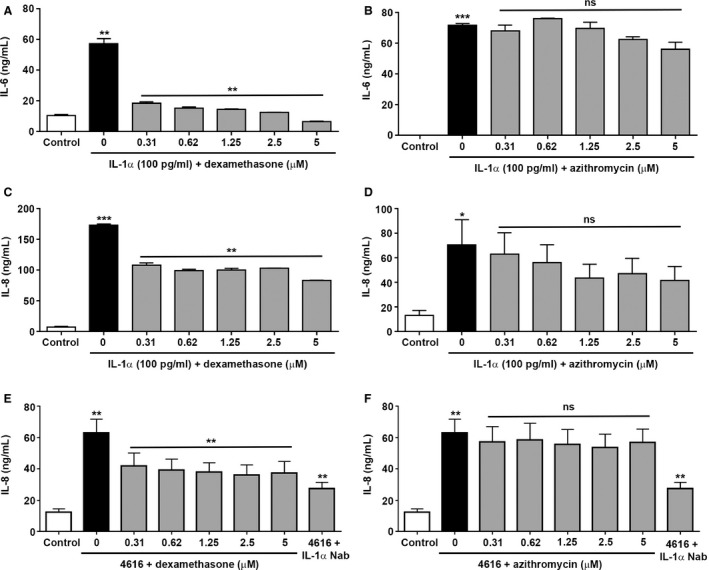
**Influence of dexamethasone and azithromycin on IL‐1α induced IL‐8 and IL‐6 protein secretion by human lung fibroblasts** ***in vitro.*** Primary lung fibroblasts (PLF) were treated with the indicated concentrations of dexamethasone (A, C, and E) or azithromycin (B, D, and F) for 1 or 24 h, respectively, after which IL‐1α (100 pg/mL) (n = 3) or media from primary lung epithelial cells (PLEC) challenged with live *Pseudomonas aeruginosa* strain 4616 (4616) (n = 6) was added and the cells were incubated for 24 h. To investigate the role of IL‐1α, the conditioned media from PLEC was pre‐incubated for 1 h with IL‐1α (4 μg/mL) neutralizing antibody. Secretion of IL‐6 (A and B) and IL‐8 (C–F) was quantified. Data were analyzed using paired t‐tests and presented as mean ± standard error of the mean. *p < 0.05, **p < 0.01, ***p < 0.001.

Next we determined whether therapeutic approaches commonly used in lung transplant recipients, namely, corticosteroids and macrolide antibiotics, would inhibit the proinflammatory phenotype of PLF driven by IL‐1α. Pretreatment of fibroblasts with increasing doses of dexamethasone significantly decreased the IL‐1α induced secretion of IL‐6 and IL‐8 (Figures 6A and C). In contrast, pretreatment of fibroblasts with increasing doses of azithromycin had no significant effect on the secretion of IL‐6 and IL‐8 (Figures 6B and D). Dexamethasone, but not azithromycin, exhibited a similar inhibitory effect on IL‐8 secretion from PLF stimulated with conditioned media from PLEC challenged with live *P. aeruginosa* (4616) (Figures 6E and F).

## Discussion

Our understanding of risk factors that contribute to the development of CLAD, particularly BOS, has increased over the last 10 years. In addition, the impact of azithromycin therapy in improving and protecting lung function in a proportion of patients developing CLAD has been clearly shown 39, 40. However, there remains a lack of therapeutic options other than retransplantation for recipients with an azithromycin nonresponsive progressive decline in lung function due to BOS.

The association between *P. aeruginosa* in the lung allograft and an increased risk of BOS has been demonstrated in numerous studies. A study by Gregson et al showed that the likelihood of transition from transplant to BOS was increased by the interaction between *Pseudomonas* and CXCL1 and that the risk of moving from transplant to death was associated with *Pseudomonas* infection 14. Dickson et al have demonstrated that symptomatic transplant recipients have a higher prevalence of *P. aeruginosa* whereas asymptomatic transplant recipients have a higher prevalence of *P. fluorescens* 13. Our group and others have shown that *de novo* acquisition of *P. aeruginosa* in recipients who were not infected pretransplant is a risk factor for development of BOS, whereas those re‐infected after transplant, probably from an upper airway reservoir, did not have an increased risk of BOS compared to those free of *P. aeruginosa* 6, 12. A study by Vos et al showed a similar association between presence of *P. aeruginosa* in the lung allograft but in contrast showed that this association was strongest in recipients with cystic fibrosis 7. Patients with cystic fibrosis are much more likely to be infected with *P. aeruginosa* prior to transplant than other groups. These conflicting results indicate that the mechanisms by which this organism increases the risk of BOS require evaluation.

In this study, we show that IL‐1α, but not HMGB1, is present in higher concentrations in BAL from lung transplant recipients developing BOS than those who do not develop BOS within 3 years of transplant. Furthermore, we show that the IL‐1α concentrations are highest within 3 months of BOS diagnosis and correlate with BAL IL‐8 levels and neutrophilia. However, whether the elevation in IL‐1α around the time of BOS diagnosis is an important factor contributing to disease pathology or is simply a consequence of BOS development requires further investigation. Additionally, IL‐1α concentration was higher in BAL samples with positive cultures for *P. aeruginosa* compared to culture negative samples or samples positive for other organisms. A potential limitation to the interpretation of our data is the lack of data regarding the presence or absence of viruses in our patient cohort. Future studies investigating the association between the levels of IL‐1α and viral infection in the development of BOS would be of great interest. The lack of a difference in HMGB1 levels in patients with BOS in our study is in agreement with data from Saito et al, who also reported no differences in HMGB1 although other alarmins such as S100A8 were found to be elevated in patients with BOS 41.

Epithelial injury has been recognized as a key event in the pathogenesis of BOS in both animal models and human histological studies 13, 26, 42, 43. However, the mechanism by which epithelial injury leads to chronic inflammation and fibrotic remodeling in the small and medium sized airways has not been elucidated. Specifically, the role of alarmins such as IL‐1α in the development of BOS has not been previously established. Our study demonstrates that infection of airway epithelium with *P. aeruginosa* causes cell injury and death and that the alarmins released are sufficient to activate lung fibroblasts to adopt a potent pro‐inflammatory phenotype *in vitro*. Furthermore, we show that IL‐1α is the major alarmin released from the damaged epithelial cells and that it is the key factor responsible for the activation of the inflammatory fibroblast phenotype *in vitro*.

Although our *in vitro* data demonstrate clearly the importance of IL‐1α released from infected epithelial cells in activating lung fibroblasts, it is possible that in the complex milieu of the transplanted lung, other cells may act as a source of IL‐1α and that other alarmins may contribute to fibroblast activation. For example, although our *in vitro* model has the benefit of using primary airway epithelial cells and primary lung fibroblasts as well as clinically isolated *Pseudomonas* strains, it is limited by the absence of immune cells or direct cell‐to‐cell contact. Therefore, another potential mechanism inducing BOS after transplantation or bacterial infection may be activation of the adaptive immune system by alarmins released by damaged cells. IL‐1 family members have previously been shown to be involved in activation of Th1 and Th17 responses 44. Additionally, in a previous study, accumulation of dendritic cells in the lung in response to cigarette smoke was shown to be IL‐1α dependent 45. The relationship between innate and adaptive immunity has been described in detail in several review articles 20, 46, 47, 48.

Safe and effective anti‐inflammatory treatment strategies for lung transplant patients are an important consideration. Interestingly, our study demonstrated that inflammation from lung fibroblasts induced by IL‐1α could not be reduced by azithromycin, which is commonly used to treat BOS. It is estimated that azithromycin is effective in approximately 50% of posttransplant patients who develop BOS. Significantly, a recent study from our group has demonstrated that lung transplant patients with persistent airway neutrophilia despite azithromycin treatment have significantly elevated levels of IL‐1α in their BAL compared to other phenotypes of CLAD including azithromycin reversible allograft dysfunction 49. The results described in the current study suggest that the epithelial alarmin IL‐1α may play an important role in contributing to chronic inflammation and the development of BOS in the transplanted lung. As IL‐1α driven inflammatory activation of fibroblasts could be partially reduced by dexamethasone, anti‐IL‐1α compounds could be a potential anti‐inflammatory therapy for transplant patients who developed BOS that is unresponsive to azithromycin.

## Disclaimer

The views expressed are those of the author(s) and not necessarily those of the NHS, the NIHR, or the Department of Health.

## Disclosure

The authors of this manuscript have no conflicts of interest to disclose as described by the *American Journal of Transplantation*.

## Supporting information


**Figure S1: Neutrophil numbers are elevated at the time of BOS diagnosis.** (A) Mean IL‐1α (i) and HMGB1 (iv) concentration and neutrophil percentage (ii) and number (iii) in BAL of lung transplant recipients who develop BOS within 3 years of transplant (n = 25). BAL were grouped into BAL samples taken >3 months before or after BOS diagnosis (>3 months before BOS) and BAL samples taken <3 months before or after BOS diagnosis (<3 months before BOS). Matching patient samples are shown with a connecting line. Mean HMGB1 (B) concentrations and neutrophil number (C) in BAL of lung transplant recipients who remained stable at 3 years (n = 25) or develop BOS within 3 years of transplant (n = 25). BAL from patients who developed BOS were grouped into BAL samples taken >3 months before or after BOS diagnosis and BAL samples taken <3 months before or after BOS diagnosis. Data were analyzed using Mann–Whitney U test or paired t‐tests as appropriate and are presented as median. Correlation between the relative time from BOS diagnosis and HMGB1 (D) concentration and neutrophil number (E) in BAL samples from patients who develop BOS. Data were analyzed using a multiple linear regression model with varying intercept. All p‐values relate to the gradient of the fitted line. To plot an average line, we took the mean value at T0 (time of BOS diagnosis) as the y‐intercept. **p < 0.01, ***p < 0.001, ****p < 0.0001. BAL, bronchoalveolar lavage; BOS, bronchiolitis obliterans syndrome; HMGB1, high mobility group protein B1.
**Figure S2: Increased neutrophilia, but not HMGB1, in BAL of culture positive posttransplant patients who develop BOS.** Mean HMGB1 (A) concentration and neutrophil number (B) in BAL from culture positive (any organism) and culture negative (no organisms) patients who developed BOS (BOS) (n = 25) or remained stable (non‐BOS) (n = 25). Mean neutrophil number in culture negative (no organisms), culture positive for any organism other than *Pseudomonas aeruginosa* (other organisms), and culture positive for *P. aeruginosa* (*P. aeruginosa*) BAL samples from patients who remained stable (non‐BOS) (C) and patients who developed BOS (D). Data were analyzed using Mann–Whitney U test and are presented as median. *p < 0.05, **p < 0.01, ***p < 0.001. BAL, bronchoalveolar lavage; BOS, bronchiolitis obliterans syndrome; HMGB1, high mobility group protein B1.
**Figure S3: Elevated neutrophilia in culture positive lung transplant recipients around the time of BOS diagnosis.** Correlation between the relative time from BOS diagnosis and HMGB‐1 (A) concentration and neutrophil percentage (B) and number in (C) culture negative (i) and culture positive (ii) BAL samples. Data were analyzed using a multiple linear regression model with varying intercept. All p‐values relate to the gradient of the fitted line. To plot an average line, we took the mean value at T0 (time of BOS diagnosis) as the y‐intercept. BOS, bronchiolitis obliterans syndrome.
**Figure S4: No correlation between IL‐1α levels and time from transplant.** Correlation between the number of months after transplantation and IL‐1α (A) and HMGB1 (B) concentrations and neutrophil percentage (C) and number (D) in BAL samples from non‐BOS patients. Data were analyzed using a multiple linear regression model with varying intercept. All p‐values relate to the gradient of the fitted line. To plot an average line, we took the mean value at T6 (time of transplant was taken as T0) as the y‐intercept. BAL, bronchoalveolar lavage; BOS, bronchiolitis obliterans syndrome; HMGB1, high mobility group protein B1.
**Figure S5: IL‐1α is elevated in BOS patients with more than one culture positive BAL sample.** Non‐BOS and BOS patients were divided into those with no positive BAL cultures, those with one positive BAL culture, and those with more than one positive BAL culture, and the levels of IL‐1α (A and B) and HMGB1 (E and F) and the percentage of neutrophils (C and D) assessed. Data were analyzed using Mann–Whitney U test and are presented as median. *p < 0.05. BAL, bronchoalveolar lavage; BOS, bronchiolitis obliterans syndrome; HMGB1, high mobility group protein B1.

**Table S1:** Primer sequences. Forward and reverse sequences for all primers used in this study.
**Table S2:** BAL organism data. Percentage and number of BAL with organisms cultured. Differences between the BOS and non‐BOS patient groups were analyzed using chi‐square test for trend test. BAL, bronchoalveolar lavage; BOS, bronchiolitis obliterans syndrome.
**Table S3:** Patients with multiple organisms. Number of BAL samples positive for more than one organism and the combination of organisms. Differences between the BOS and non‐BOS patient groups were analyzed using chi‐square test for trend test. BAL, bronchoalveolar lavage; BOS, bronchiolitis obliterans syndrome.
**Table S4:** Time of colonization. Number of positive BAL samples relative to time from transplant or time from BOS diagnosis. BAL, bronchoalveolar lavage; BOS, bronchiolitis obliterans syndrome.

**Data S1:** Supplementary Materials and Methods.
