# Supplementary material for: Pseudomonas aeruginosa Induced Airway Epithelial Injury Drives Fibroblast Activation: A Mechanism in Chronic Lung Allograft Dysfunction
Source: Am J Transplant. 2016 Feb 26;16(6):1751–65. doi: 10.1111/ajt.13690 (PMC4879508; doi:10.1111/ajt.13690)

Supplementary figure 1

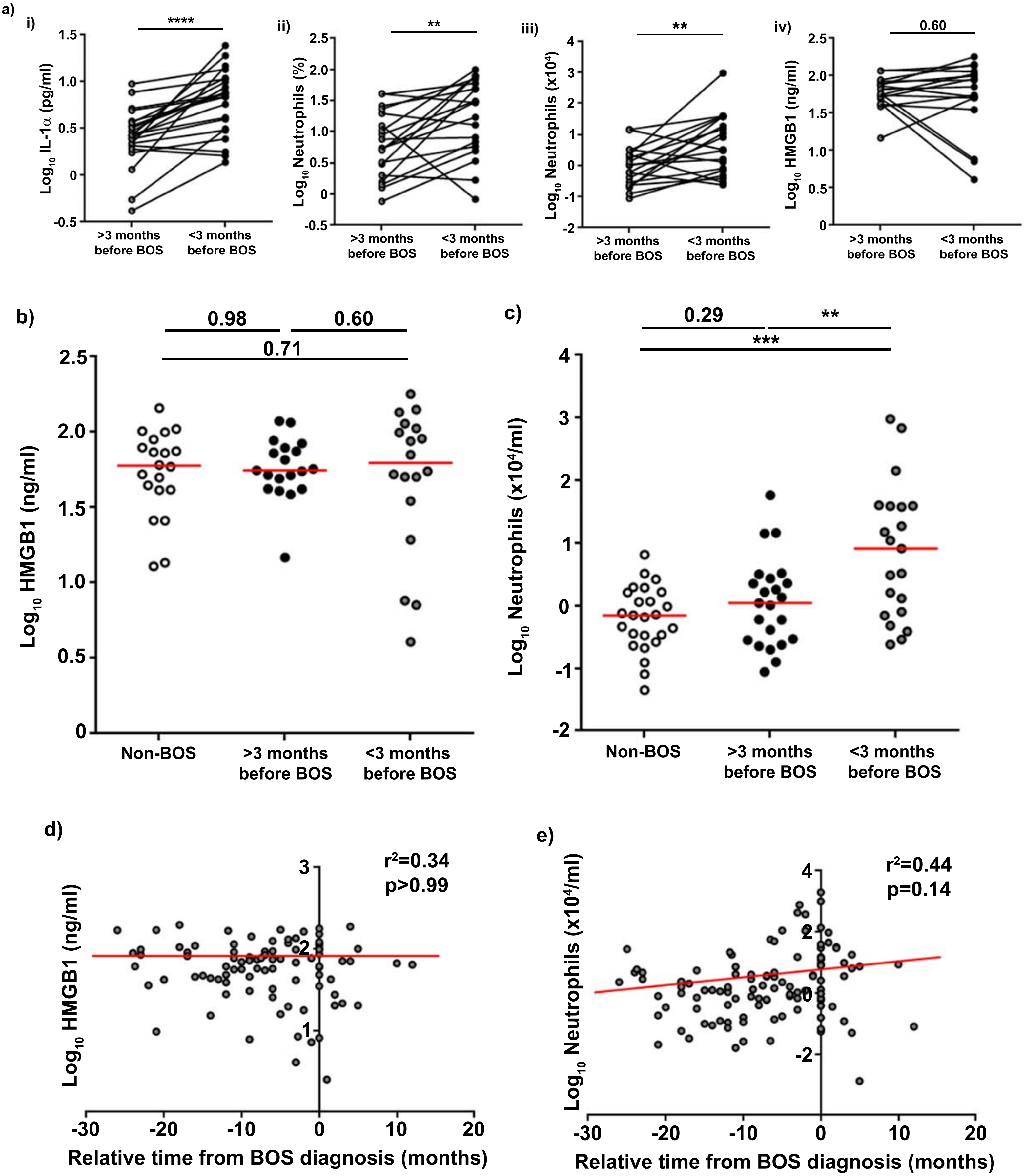

Supplementary figure 2

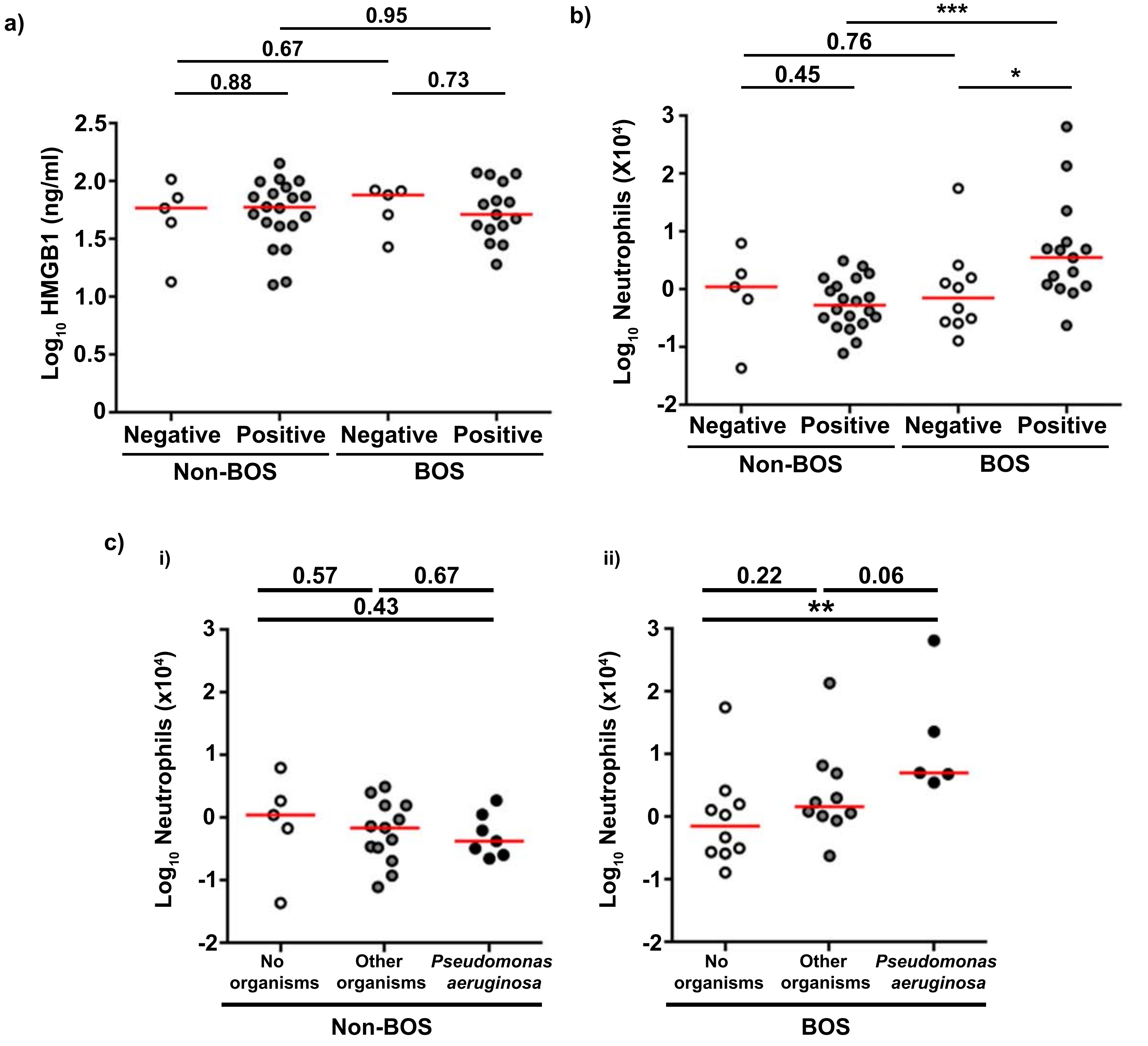

Supplementary figure 3

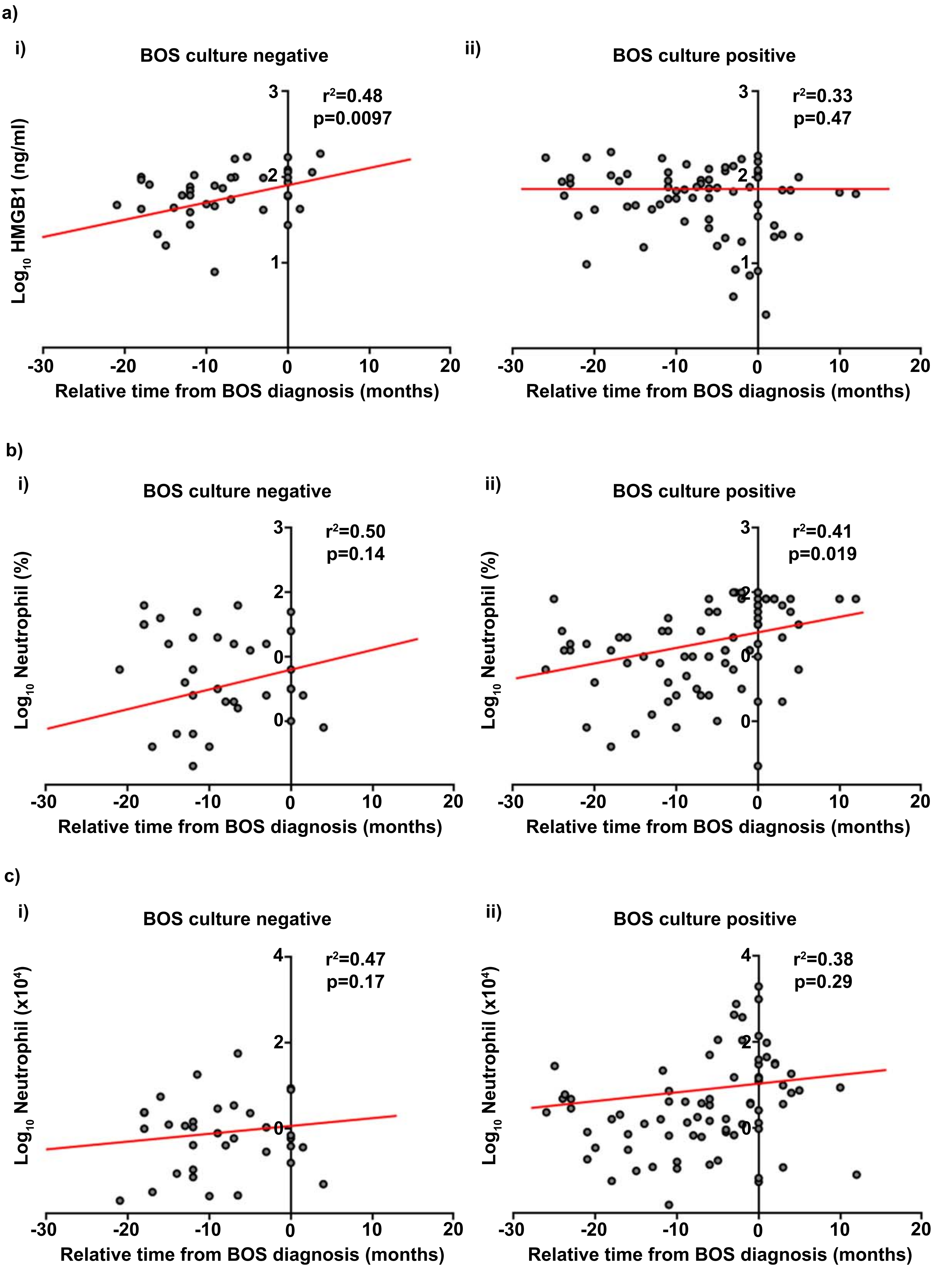

Supplementary figure 4

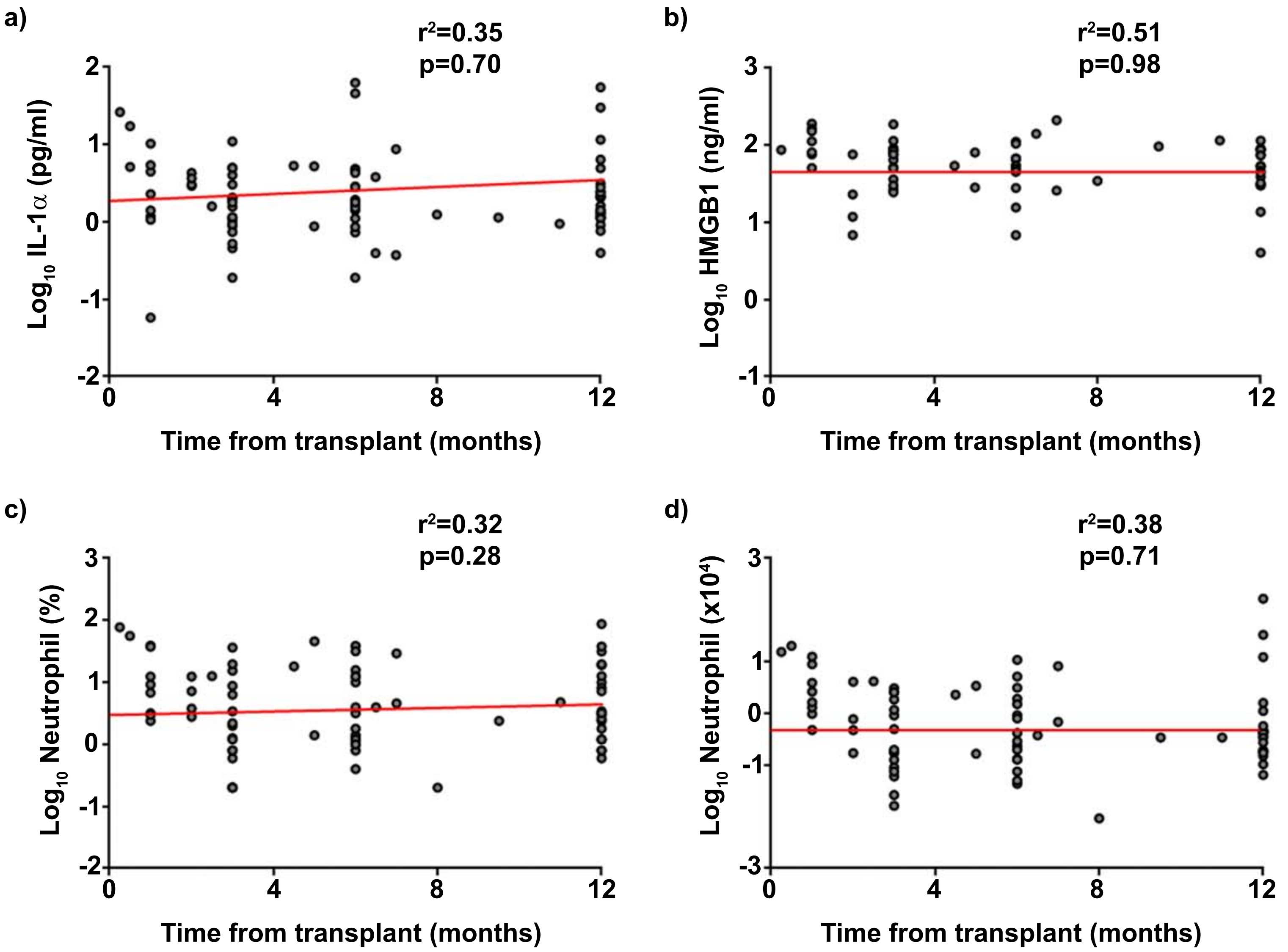

Supplementary figure 5

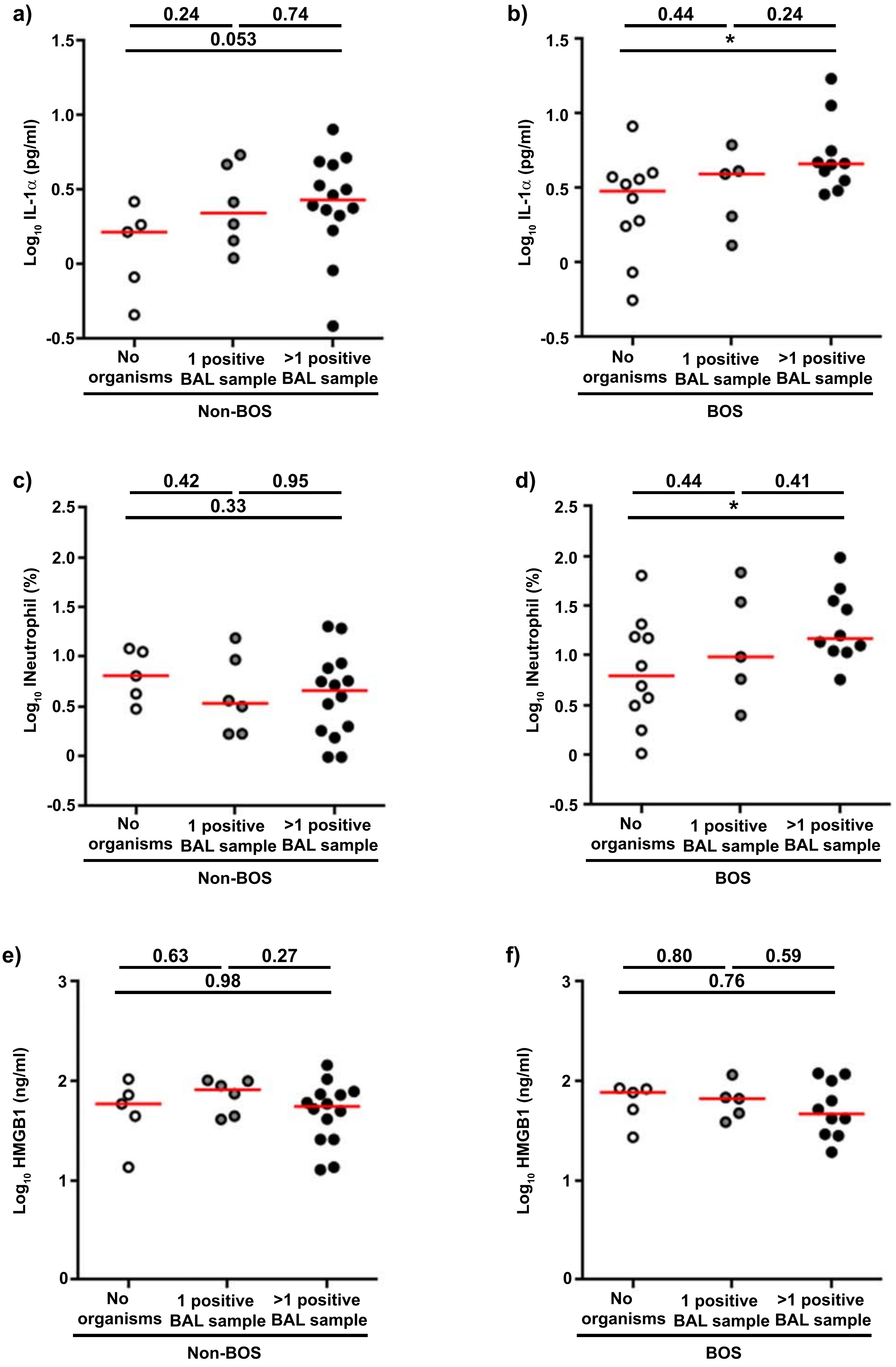

Supplement: Supplementary file 1 — Figure S1: Neutrophil numbers are elevated at the time of BOS diagnosis. (A) Mean IL‐1α (i) and HMGB1 (iv) concentration and neutrophil percentage (ii) and number (iii) in BAL of lung transplant recipients who develop BOS within 3 years of transplant (n = 25). BAL were grouped into BAL samples taken >3 months before or after BOS diagnosis (>3 months before BOS) and BAL samples taken <3 months before or after BOS diagnosis (<3 months before BOS). Matching patient samples are shown with a connecting line. Mean HMGB1 (B) concentrations and neutrophil number (C) in BAL of lung transplant recipients who remained stable at 3 years (n = 25) or develop BOS within 3 years of transplant (n = 25). BAL from patients who developed BOS were grouped into BAL samples taken >3 months before or after BOS diagnosis and BAL samples taken <3 months before or after BOS diagnosis. Data were analyzed using Mann–Whitney U test or paired t‐tests as appropriate and are presented as median. Correlation between the relative time from BOS diagnosis and HMGB1 (D) concentration and neutrophil number (E) in BAL samples from patients who develop BOS. Data were analyzed using a multiple linear regression model with varying intercept. All p‐values relate to the gradient of the fitted line. To plot an average line, we took the mean value at T0 (time of BOS diagnosis) as the y‐intercept. **p < 0.01, ***p < 0.001, ****p < 0.0001. BAL, bronchoalveolar lavage; BOS, bronchiolitis obliterans syndrome; HMGB1, high mobility group protein B1. Figure S2: Increased neutrophilia, but not HMGB1, in BAL of culture positive posttransplant patients who develop BOS. Mean HMGB1 (A) concentration and neutrophil number (B) in BAL from culture positive (any organism) and culture negative (no organisms) patients who developed BOS (BOS) (n = 25) or remained stable (non‐BOS) (n = 25). Mean neutrophil number in culture negative (no organisms), culture positive for any organism other than Pseudomonas aeruginosa (other organisms [file AJT-16-1751-s001.pdf]
