# Supplementary material for: Pseudomonas aeruginosa Induced Airway Epithelial Injury Drives Fibroblast Activation: A Mechanism in Chronic Lung Allograft Dysfunction
Source: Am J Transplant. 2016 Feb 26;16(6):1751–65. doi: 10.1111/ajt.13690 (PMC4879508; doi:10.1111/ajt.13690)
Supplement: Supplementary file 2 — Table S1: Primer sequences. Forward and reverse sequences for all primers used in this study. Table S2: BAL organism data. Percentage and number of BAL with organisms cultured. Differences between the BOS and non‐BOS patient groups were analyzed using chi‐square test for trend test. BAL, bronchoalveolar lavage; BOS, bronchiolitis obliterans syndrome. Table S3: Patients with multiple organisms. Number of BAL samples positive for more than one organism and the combination of organisms. Differences between the BOS and non‐BOS patient groups were analyzed using chi‐square test for trend test. BAL, bronchoalveolar lavage; BOS, bronchiolitis obliterans syndrome. Table S4: Time of colonization. Number of positive BAL samples relative to time from transplant or time from BOS diagnosis. BAL, bronchoalveolar lavage; BOS, bronchiolitis obliterans syndrome. [file AJT-16-1751-s002.pdf]

Supplementary Table 1

Gene name	Forward primer sequence	Reverse primer sequence
GM-CSF	CATGATGGCCAGCCACTAC	AATCTGGGTTGCACAGGAAG
IL-8	GTGGAGTTTTGCCAAGGAGT	CTCTGCACCCAGTTTTCCTT
MMP3	GCAGTTTGCTCAGCCTATCC	GAGTGTCGGAGTCCAGCTTC
GAPDH	GAGTCAACGGATTTGGTCGT	GACAAGCTTCCCGTTCTCAG

Supplementary table 1 – Primer sequences

Supplementary table 2

Organisms cultured from BAL	BOS		Non-BOS		p-value
	Number of BAL	% of BAL	Number of BAL	% of BAL	
Pseudomonas aeruginosa	19	16%	13	14%	p=0.36
Candida albicans	13	11%	18	20%	
Aspergillus fumigatus	6	5%	6	7%	
Proteus mirabilis	4	3%	0	0%	
Stenotrophomonas maltophilia	4	3%	0	0%	
Staph aureus	2	2%	4	4%	
Acinetobacter baumannii	1	1%	0	0%	
Aspergillus nidulans	1	1%	0	0%	
Enterobacter cloacae	1	1%	0	0%	
Haemophilus influenza	1	1%	0	0%	
Klebsiella pneumonia	1	1%	0	0%	
Serratia sp	1	1%	1	1%	
MRSA	0	0%	7	8%	
E. Coli	0	0%	2	2%	
Burkholderia cepacia complex	0	0%	1	1%	
Exophiala sp	0	0%	1	1%	
No organisms cultured	73	62%	46	51%	

Supplementary table 2 – BAL organism data

Supplementary Table 3

	BOS		non-BOS		
Organisms cultured from BAL	Number	%	Number	%	p-value
Aspergillus fumigatus & Aspergillus nidulans	1	4%	0	0%	p=0.60
Aspergillus fumigatus & Candida albicans	1	4%	1	4%	
Aspergillus fumigatus & Candida albicans & Proteus mirabilis	1	4%	0	0%	
Aspergillus fumigatus & Pseudomonas aeruginosa	1	4%	0	0%	
Candida albicans & Pseudomonas aeruginosa	1	4%	2	8%	
Candida albicans & Staph aureus	1	4%	1	4%	
Klebsiella pneumonia & Stenotrophomonas maltophilia	1	4%	0	0%	
Pseudomonas aeruginosa & Stenotrophomonas maltophilia	1	4%	0	0%	
Candida albicans & MRSA	0	0%	1	4%	
Candida albicans & Serratia sp	0	0%	1	4%	
Exophiala sp & Pseudomonas aeruginosa	0	0%	1	4%	

Supplementary Table 3 – Patients with multiple organisms

Supplementary Table 4

	Number of samples		
**Time of BAL**	**Number**	**Positive**	**%**
<3 months before or after BOS diagnosis	41	15	37%
Between 3-6 month before BOS diagnosis	12	8	67%
Between 6-12 month before BOS diagnosis	29	9	31%
>12 month before BOS diagnosis	27	8	30%
>3 months after BOS diagnosis	8	4	50%

	Number of samples		
**Time of BAL**	**Number**	**Positive**	**%**
<3 months after transplant	35	21	60%
3-6 month after transplant	20	11	55%
>6 months after transplant	35	12	34%

Supplementary Table 4 – Time of colonisation
