# Supplementary material for: Pseudomonas aeruginosa Induced Airway Epithelial Injury Drives Fibroblast Activation: A Mechanism in Chronic Lung Allograft Dysfunction
Source: Am J Transplant. 2016 Feb 26;16(6):1751–65. doi: 10.1111/ajt.13690 (PMC4879508; doi:10.1111/ajt.13690)
Supplement: Supplementary file 3 — Data S1: Supplementary Materials and Methods. [file AJT-16-1751-s003.doc]

**Supplementary materials and methods**

**Cell culture**

Primary human lung epithelial cells (PLEC) and primary human lung fibroblasts (PLF) were cultured from human donor lungs not meeting acceptance criteria for lung transplantation due to poor function as a result of focal consolidation or focal lung contusion. Cells were harvested from an area of normal lung distant to the focal changes that prevented their use.

PLEC were harvested by bronchial brushings from ~2 to 5mm airways and established on collagen coated surface in Airway Epithelial Cell medium (C-21160, Promocell) supplemented with 100U/ml penicillin and 100μg/ml streptomycin. Epithelial phenotype was confirmed by positive expression of E-cadherin, ZO-1 and negligible expression of fibronectin, vimentin, α-smooth muscle actin and CD45.

Primary human lung fibroblasts (PLF) were isolated using a collagenase II digestion technique. Briefly, lung tissue pieces were minced, washed on a 40μm filter with DMEM/F12 (Sigma) and digested with 0.2% type II collagenase (Worthington Biochemical Corporation) at room temperature for 2 hours. After washing with PBS the cells were filtered through large gauze followed by a 100μm filter. The cells were collected by centrifugation at 300xg and plated at a density of 80,000 cells/cm^2^ in DMEM/F12 (Sigma) supplemented with 10% FBS, 1% L-glutamine, 100U/ml penicillin and 100μg/ml streptomycin. The cells were left to attach for 1 hour, after which they were washed with 1xPBS and fresh culture medium was added. Mesenchymal phenotype was confirmed by positive expression of fibronectin, vimentin and α-smooth muscle actin and negligible expression of E-cadherin, ZO-1 and CD45.

**Bacterial culture**

*Pseudomonas aeruginosa* strains were cultured from the sputa of patients post lung transplantation. Bacteria were routinely grown in Tryptone Soy Broth (TSB) at 37°C with shaking or on Tryptone Soy Agar (TSA) at 37°C where appropriate. For addition to epithelial cells, bacteria were grown for 16-18 hours in TSB at 37°C with shaking. Bacterial cultures were adjusted to an optical density of 1 at 600nm (~10^9^cfu/ml) and washed with PBS before resuspension in airway epithelial media with no antibiotics. Bacteria were subsequently diluted to 10^4^ or 10^5^ cfu/ml as required. For preparation of heat killed bacteria, a suspension of bacteria at 10^9^ cfu/ml in antibiotic-free airway epithelial media was incubated at 65°C for 45 min. In each case, preparations were confirmed to be free of viable bacteria by plating aliquots onto TSA and incubating at 37°C for 24 hours.

**HMGB-1 ELISA**

Neat sample or hrHMGB1(100μl)(R&D Systems) were added to a 96 well plate and incubated overnight at room temperature. Plates were blocked with 3%BSA/PBS and antigen detected using 2µg/ml of anti-human HMGB1 antibody(MAB1690, R&D Systems) followed by an anti-mouse IgG biotinylated antibody(1:2000)(BAF018, R&D systems). Plates were developed with substrate reagent(DY999, R&D Systems) and the reactions stopped with 1M H_2_SO_4_. Optical density was measured at 450nm. HMGB1 concentration was calculated against a seven point standard curve prepared by 2-fold dilutions of hrHMGB1 with a high concentration of 1µg/ml.
