# Supplementary material for: Pseudomonas aeruginosa Induced Airway Epithelial Injury Drives Fibroblast Activation: A Mechanism in Chronic Lung Allograft Dysfunction
Source: Am J Transplant. 2016 Feb 26;16(6):1751–65. doi: 10.1111/ajt.13690 (PMC4879508; doi:10.1111/ajt.13690)
Supplement: Supplementary file 4 [file AJT-16-1751-s004.doc]

**Supplemental figure legends**

**Supplementary figure 1 - Neutrophil numbers are elevated at the time of BOS diagnosis**

(a) Mean IL-1α (i) and HMGB1 (iv) concentration and neutrophil percentage (ii) and number (iii) in BAL of lung transplant recipients who develop BOS within 3 years of transplant (n=25). BAL were grouped into BAL samples taken >3 months before or after BOS diagnosis (>3 months before BOS) and BAL samples taken <3 months before or after BOS diagnosis (<3 months before BOS). Matching patient samples are shown with a connecting line. Mean HMGB1 (b) concentrations and neutrophil number (c) in BAL of lung transplant recipients who remained stable at 3 years (n=25) or develop BOS within 3 years of transplant (n=25). BAL from patients who developed BOS were grouped into BAL samples taken >3 months before or after BOS diagnosis and BAL samples taken <3 months before or after BOS diagnosis. Data was analyzed using Mann-Whitney U Test or paired T-tests as appropriate and is presented as median. Correlation between the relative time from BOS diagnosis and HMGB-1 (d) concentration and neutrophil number (e) in BAL samples from patients who develop BOS. Data was analyzed using a multiple linear regression model with varying intercept. All p-values relate to the gradient of the fitted line. To plot an average line, we took the mean value at T0 (time of BOS diagnosis) as the y-intercept. **p<0.01, ***p<0.001, ****p<0.0001.

**Supplemental figure 2 - Increased neutrophilia, but not HMGB1, in BAL of culture positive post-transplant patients who develop BOS**

Mean HMGB1 (a) concentration and neutrophil number (b) in BAL from culture positive (any organism) and culture negative (no organisms) patients who developed BOS (BOS)(n=25) or remained stable (non-BOS)(n=25). Mean neutrophil number in culture negative (no organisms), culture positive for any organism other than *Pseudomonas aeruginosa* (other organisms) and culture positive for *Pseudomonas aeruginosa* (*Pseudomonas aeruginosa*) BAL samples from patients who remained stable (non-BOS) (c ) and patients who developed BOS (d). Data was analyzed using Mann-Whitney U Test and is presented as median. *p<0.05, **p<0.01, ***p<0.001.

**Supplementary figure 3 – Elevated neutrophilia in culture positive lung transplant recipients around the time of BOS diagnosis**

Correlation between the relative time from BOS diagnosis and HMGB-1 (a) concentration and neutrophil percentage (b) and number in (c) in culture negative (i) and culture positive (ii) BAL samples. Data was analyzed using a multiple linear regression model with varying intercept. All p-values relate to the gradient of the fitted line. To plot an average line, we took the mean value at T0 (time of BOS diagnosis) as the y-intercept.

**Supplementary figure 4 – No correlation between IL-1α levels and time from transplant**

Correlation between the number of months after transplantation and IL-1α (a) and HMGB-1 (b) concentrations and neutrophil percentage (c) and number in BAL (d) in BAL samples from non-BOS patients. Data was analyzed using a multiple linear regression model with varying intercept. All p-values relate to the gradient of the fitted line. To plot an average line, we took the mean value at T6 (time of transplant was taken as T0) as the y-intercept.

**Supplementary figure 5 – IL-1α is elevated in BOS patients with >1 culture positive BAL sample**

Non-BOS and BOS patients were divided into those with no positive BAL cultures, those with 1 positive BAL culture and those with >1 positive BAL culture and the levels of IL-1α (a & b) &HMGB1 (e & f) and the percentage of neutrophils (c & d) assessed. Data was analyzed using Mann-Whitney U Test and is presented as median. *p<0.05.

**Supplementary table 1 – Primer sequences**

Forward and reverse sequences for all primers used in this study.

**Supplementary table 2 - BAL organism data**

Percentage and number of BALs with organisms cultured. Differences between the BOS and non-BOS patient groups were analysed using chi-square test for trend test.

**Supplementary table 3 – Patients with multiple organisms**

Number of BAL samples positive for >1 organism and the combination of organisms. Differences between the BOS and non-BOS patient groups were analysed using chi-square test for trend test.

**Supplementary table 4 – Time of colonisation**

Number of positive BAL samples relative to time from transplant or time from BOS diagnosis.
